# Dispersion of nonresonant third-order nonlinearities in GeSiSn ternary alloys

**DOI:** 10.1038/srep32622

**Published:** 2016-09-13

**Authors:** Francesco De Leonardis, Benedetto Troia, Richard A. Soref, Vittorio M. N. Passaro

**Affiliations:** 1 Photonics Research Group, Dipartimento di Ingegneria Elettrica e dell’Informazione, Politecnico di Bari Via Edoardo Orabona n. 4, 70125 Bari, Italy; 2 Department of Engineering, The University of Massachusetts, Boston, Massachusetts, 02125, USA

## Abstract

Silicon (Si), tin (Sn), and germanium (Ge) alloys have attracted research attention as direct band gap semiconductors with applications in electronics and optoelectronics. In particular, GeSn field effect transistors can exhibit very high performance in terms of power reduction and operating speed because of the high electron drift mobility, while the SiGeSn system can be constructed using CMOS-compatible techniques to realize lasers, LED, and photodetectors. The wide Si, Ge and Sn transparencies allow the use of binary and ternary alloys extended to mid-IR wavelengths, where nonlinearities can also be employed. However, neither theoretical or experimental predictions of nonlinear features in SiGeSn alloys are reported in the literature. For the first time, a rigorous and detailed physical investigation is presented to estimate the two photon absorption (TPA) coefficient and the Kerr refractive index for the SiGeSn alloy up to 12 μm. The TPA spectrum, the effective TPA wavelength cut-off, and the Kerr nonlinear refractive index have been determined as a function of alloy compositions. The promising results achieved can pave the way to the demonstration of on-chip nonlinear-based applications, including mid-IR spectrometer-on-a-chip, all-optical wavelength down/up-conversion, frequency comb generation, quantum-correlated photon-pair source generation and supercontinuum source creation, as well as Raman lasing.

Group-IV photonics refers to manufacturable opto-electronic integrated circuits (OEICs) upon an SOI substrate. In this scenario, although the integration of active optoelectronic components on chip can comprise both hybrid and monolithic technological approaches, total monolithic integration of group IV materials is expected over the long term to lower the costs of photonics integrated circuits (PICs) and to enhance their reliability with respect to PICs based on hybrid III-V-on-Si platforms^1^. Silicon (Si) and germanium (Ge) group-IV semiconductors have been widely used for advanced linear and nonlinear photonic applications. However, the Si and Ge indirect bandgaps represent a drawback when these materials are used for realizing active devices for optoelectronic applications. Indeed, momentum conservation is not satisfied in the first order process involving one photon and, therefore, stimulated emission of photons is not as efficient as that achievable in III-V components. In this context, SiGeSn heterostructures have been demonstrated to be suitable for advancing monolithic integration of photonic active devices because they allow a complete suite of active on-chip photonic components^1^. In the last few years, despite the extensive scientific studies on the binary crystalline Ge_1−y_Sn_y_ alloy, the Ge_1−x−y_Si_x_Sn_y_ ternary alloy has not yet received a strong interest in the scientific community, although it deserves additional investigations^1^,^2^. Indeed, Ge_1−x−y_Si_x_Sn_y_ exhibits more thermodynamic stability than the photonic/optoelectronic devices made from Ge_1__−y_Sn_y_^3^,^4^,^5^,^6^,^7^,^8^,^9^.

A theoretical model for estimating the optical gain of a strained Ge_1−x_Si_x_/Si_x_Ge_y_Sn_1−x−y_ quantum-well (QW) structure has been reported in ref. 10. In particular, a tensile strained Ge QW with a direct band gap for the electron and hole confinements has been realized using the ternary alloy as the barriers. Additionally, reasonable material parameters have been used to estimate the transition energy, the optical gain spectrum, and the effects of the carrier leakage in the presence of the quantized sub-bands. Recently, a strain-balanced Ge_z_Sn_1−z_/Si_x_Ge_y_Sn_1−x−y_ multiple-quantum-well (MQW) laser has also been proposed^11^, where a proper amount of α-Sn has been used into Ge to achieve the population inversion in the direct conduction band. However, the Ge_1−x−y_Si_x_Sn_y_ ternary alloy can guarantee a better flexibility in realizing heterostructures. Indeed, it is possible to explore the design space for heterostructures in order to realize QWs based on strain-free (lattice-matched) pairs of Ge_1−x−y_Si_x_Sn_y_ alloys, one of which would act as a QW and the other as the barrier material. In this sense, Ge_1−x−y_Si_x_Sn_y_ MQW lasing has been assumed as feasible within the wavelength range of 2.2–6.0 μm^12^,^13^. Moreover, looking beyond midwave, there are a number of longwave, and far infrared opportunities for using the Ge_1−x−y_Si_x_Sn_y_ alloy in order to realize intersubband quantum cascade lasers (QCL). In ref. 14, the authors propose a Ge/Ge_0.76_Si_0.19_Sn_0.05_ QCL by using inter-subband transitions at *L* valleys of the conduction band which have a clean offset of 150 meV situated below other energy valleys, *X*. The entire structure is strain-free because the lattice-matched Ge and Ge_0.76_Si_0.19_Sn_0.05_ layers are to be grown on a relaxed Ge buffer layer on a Si substrate.

Very recently, the demonstration of prototype Ge_1−x−y_Si_x_Sn_y_ light emitting diodes with distinct direct and indirect edges and high quality I-V characteristics has been reported in ref. 15. The devices were fabricated on Si(100) wafers in heterostructure p-i-n geometry [n-Ge/i-Ge_1−x−y_Si_x_Sn_y_/p-Ge(Sn/Si)], where the intrinsic layer has a Sn content ranging from 3.5% to 11%, while the Si content was kept constant at nearly 3%. The state-of-the-art briefly summarized above, confirms that the research efforts are mainly focused on the design and realization of electrically pumped lasers. However, the nonlinear photonics based on the ternary alloy Ge_1−x−y_Si_x_Sn_y_ is still an open issue.

The experimental measurements carried out on Si and Ge indicate that the Ge_1−x−y_Si_x_Sn_y_ alloy can exhibit a strong third-order nonlinear susceptibility, *χ*^(3)^, even larger than that in Si and roughly comparable to that in Ge, depending upon the operating wavelength. The experimental measurements carried out on Si and Ge indicate that the Ge_1−x−y_Si_x_Sn_y_ alloy can exhibit a strong third order nonlinear susceptibility *χ*^(3)^ even larger than that in Si and roughly comparable to that in Ge, depending upon the operating wavelength. The likelihood of strong X^(3)^ in the ternary is indicated by recent *χ*^(3)^ experimental results on Ge_1−y_Sn_y_ where a strong Franz-Keldysh effect was observed^16^. Thus, the nonlinear optical (NLO) response of Ge_1−x−y_Si_x_Sn_y_ structures could open up a wide range of applications such as four-wave mixing (FWM), wavelength conversion, third harmonic generation (THG), infrared parametric amplification, continuum generation, self-phase modulation (SPM), as well as tunable oscillation. The only caveat is the presence of two-photon absorption (TPA), which is going to be stronger in the Ge_1−x−y_Si_x_Sn_y_ ternary than in silicon material.

The design of NLO devices mentioned here and based on new technological platforms will surely rely upon knowledge of the relevant third-order NLO coefficients (i.e., TPA coefficient, *β*^TPA^, and Kerr nonlinear index *n*_2_). Furthermore, these coefficients may have strong wavelength dependences that are worth being investigated. Actually, to the best of our knowledge, the experimental knowledge of these coefficients is missing in the literature, thus we have analyzed the different approaches proposed in the literature to assist NLO design, with the aim to figure out *β*^TPA^ and Kerr nonlinear index *n*_2_ coefficients. Generally, the analytical models heavily underestimate *β*^TPA^ with respect to experimental data, thus requiring the use of fitting coefficients. To this purpose, we have addressed these problems here, proposing non-trivial physical generalizations in order to overcome the necessity of fitting parameters.

## Theoretical Background

The goal of this section is to propose a physical model in order to evaluate the theoretical wavelength dispersion for both the third-order absorption and nonlinear Kerr refractive index occurring in the ternary alloy Ge_1−x−y_Si_x_Sn_y_. In particular, our approach consists in figuring out the fundamental and dominant contributions of the TPA process, in the cases of direct and indirect transitions.

### Direct transitions

As outlined in refs 17,18, the TPA effect induced by direct transitions is mainly dominated by the allowed-forbidden (a–f) transitions. Among a number of models that describe the two photon processes in crystals^19^, we adopt and modify the three-band model proposed by Basov *et al*.^19^,^20^, where the intra-band terms are considered. The mathematical expression proposed here is a generalization of Basov’s equations because it includes the non-parabolicity and exciton effects. Essentially, the model includes one conduction band, *c*, and two valence bands, *v*_1_ (heavy hole) and *v*_2_ (light hole), that are degenerate at the wavevector **K** = 0. Then, the effective transition moment is a combination of an allowed-forbidden transition (*v* → *c*, *c* → *c*), and a forbidden-allowed transition (*v* → *v*, *v* → *c*). Also, the non-degenerate TPA coefficient induced when two optical beams at frequencies *ω*_1_ and *ω*_2_ act simultaneously, can be estimated by beginning with the transition rate as:


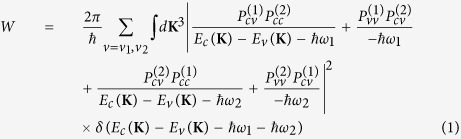


In Eq. (1), *E*_*c*,*v*_(**K**) is the energy in conduction (*c*) or valence (*v*) bands, *δ*(*E*_*c*_(**K**) − *E*_*v*_(**K**) − *ħ*;*ω*_1_ − *ħ*;*ω*_2_) is the Dirac delta function, 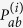 (*a*(*b*) = *c*, *v*) is the transition matrix element for an optical beam of frequency *ω*_*i*_ and polarization *e*^(*i*)^ defined as in Eq. (2), where all parameters are measured in SI units:


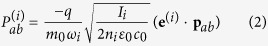


In Eq. (2), the coefficients *m*_0_, *q*, *ε*_0_, and *c*_0_ represent the electron mass at rest, the elementary charge, the vacuum permittivity and the light velocity, respectively. The terms *I*_*i*_ and *n*_*i*_ indicate the optical intensity and the semiconductor linear refractive index at *ω*_*i*_, respectively. Moreover, it is worth outlining that for dipole-allowed transitions (*a* ≠ *b*), the electron momentum matrix element is independent of **K**, and it is related to the semiconductor Kane energy by the relationship |***p***_*ab*_|^2^ = 0.5*m*_0_*E*_*p*_^18^. On the contrary, if *a* = *b* (dipole-forbidden) the matrix element is defined as **p**_*aa*_ = ±*m*_0_**K**/*m*_*a*_, where *m*_*a*_ is the effective mass of the electron, and the leading sign for conduction or valence bands is positive or negative, respectively. Consequently, by using the parabolic expression for the inter-band energy separation (*E*_*c*_(**K**) − *E*_*v*_(**K**) = *E*_*g*,*d*_ + *ħ*;^2^*k*^2^/2*m*_*cv*_, with *m*_*cv*_ the reduced effective mass), and considering the non-degenerate TPA coefficient, *β*^TPA^(*ω*_1_, *ω*_2_) = *Wħω*_1_/(2*I*_1_*I*_2_), we demonstrate Eq. (3).


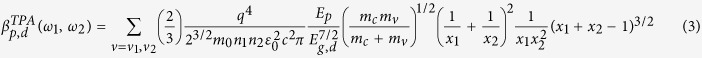


In Eq. (3), *E*_*g*,*d*_ is the semiconductor direct energy gap, and the normalized variable *x*_*i*_ is defined as *x*_*i*_ = *ħ*;*ω*_*i*_/*E*_*g*,*d*_. Moreover, in the first term of Eq. (3), the factors 2 and 3 in the ratio are related to the electron spin degeneracy and the angular average of the contributions **e**^(*i*)^ ⋅ **K**, respectively. It is worth outlining that the spectral dependence of Eq. (3) is commonly used in the literature^17^,^18^, even if the light hole contribution is generally neglected and a fitting coefficient is adopted in order to match the experimental measurements. However, if the non-parabolic form is used instead of the parabolic expression for the energy bands as a function of the wave vector, then Eqs (4) and (5) are obtained.


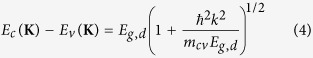



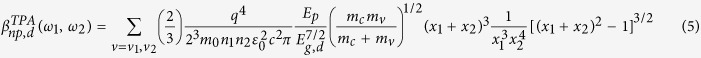


Hereafter, we use the subscripts (*p*) and (*np*) to specify the assumption of parabolic and non-parabolic bands, respectively. As shown in Eq. (5), the hypothesis of non-parabolicity produces a functional dependence from the photon energies substantially different from Eq. (3), inducing the condition 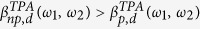.

It is worth outlining that Eqs (3) and (5) do not include the exciton effects. In this sense, it might be expected that the electron-hole Coulomb interaction could induce an enhancement of the TPA coefficient. Generally speaking, even if *x*_1_ + *x*_2_ ≥ 1, the continuum of excitons can induce an enhancement of the TPA process.

Analogously to the case of the single photon direct transition investigated by Elliot^21^, we can estimate the continuum exciton influence by multiplying Eq. (3) or (5) by the envelope function reported in Eq. (6).


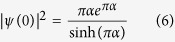


where 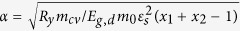 Finally, the coefficients *R*_*y*_ = 13.6 eV and *ε*_s_ represent the Rydberg energy and the semiconductor static dielectric constant, respectively.

### Indirect transitions

The investigation of the two photon transition probability when the maximum of the valence band and the minimum of the conduction band do not occur at the same point of the Brillouin zone requires a sophisticated approach.

**Figure 1:**
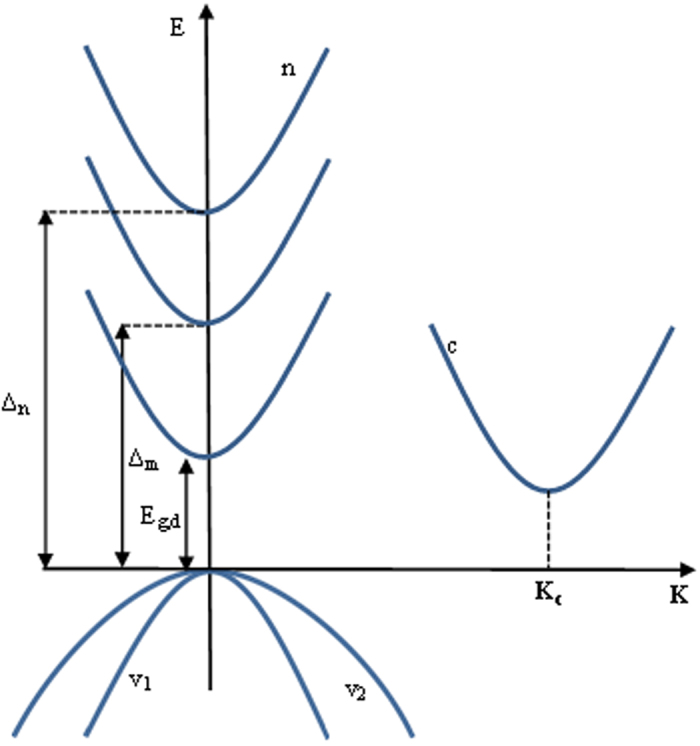
Band scheme for indirect transition with partecipation of two photons and one phonon.

The need to ensure a physical interpretation of the indirect nonlinear absorption processes occurring in the ternary alloy Ge_1−x−y_Si_x_Sn_y_ induces us to generalize the theory proposed by Bassani *et al*.^19^. In this context, we adopt a non-fitted first-principles model based on five bands as sketched in Fig. 1.

Actually, an electron makes a transition from the doubly degenerate valence bands at **K** = 0, *v*_1_ (heavy hole) and *v*_2_ (light hole), to the conduction band *c* at **K** = **K**_0_, through the intermediate states *n* and *m*. Therefore, two photons at the frequencies *ω*_1_ and *ω*_2_ are absorbed to transit from the valence bands to the intermediate states, then a phonon of energy *E*_*ph*_ = *ħ*;Ω is absorbed or emitted in order to complete the transition from one of the two intermediate states to the conduction band. Moreover, the momentum conservation involves the phonons that correspond to a wave vector − **K** in creation or **K** in destruction.

Generally speaking, the two-photons indirect absorption can also be influenced by the Coulomb interaction. In this sense, we propose a generalization of the model described in ref. 21, in which the continuum exciton influence is taken into account. Since exact calculations are very complicated, it is convenient to make some approximations in order to derive an analytical formulation for the non-degenerate TPA indirect coefficient. According to^21^, we assume that the transitions involved in the process have **K**_**v**_ ≅ 0, and **K**_**e**_ ≅ **K**_0_. Therefore, only electron and hole states that are close to extrema give an appreciable contribution and will be considered in the following. In this context, beyond the exciton bands, i.e., when *E* > 0 (see Eq. (7)), there is a further absorption from the continuum.


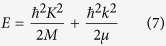


In Eq. (7), *k* is wave-vector related to the relative motion, while the masses *M* and *μ* are defined as *M* = *M*_*c*_ + *m*_*v*_ and *μ* = *M*_*c*_*m*_*v*_/(*M*_*c*_ + *m*_*v*_), respectively. In particular, since the absorption process involves indirect conduction valleys, the electron effective mass may be approximated with its density-of-states average 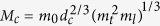, where *m*_*t*_, *m*_*l*_, and *d*_*c*_ represent the transverse, the longitudinal masses, and the number of equivalent conduction band minima, respectively^22^,^23^. Equation (8) can be obtained from the third-order time dependent perturbation theory, and it allows the transition rate to be calculated:


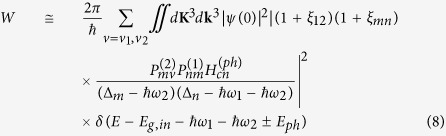


In Eq. (8), |*ψ*(0)|^2^ and *E*_*g*,*in*_ indicate the envelope function taking into account the continuum exciton influence (as specified in the following) and the indirect bandgap energy, respectively. The terms 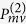 and 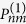 are the transition matrix element for the optical transitions 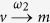, and 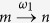, respectively. In addition, 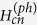
represents the electron-phonon interaction Hamiltonian satisfying the relationship 
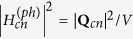
, with *V* the crystal volume and |*Q*_*cn*_|^2^ the matrix element for phonon scattering. Moreover, the term *ξ*_12_ exchanges photon 1 with photon 2. Analogously, *ξ*_*mn*_ indicates the permutation of the intermediate states. Indeed, the exchange in the order of the intermediate states can induce two different indirect transitions, (a) 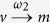, 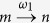, and 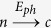, (b) 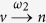, 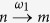, and 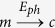. Thus, *ξ*_*mn*_ can assume a value of 0 or 1 if one or both the transitions are allowed by the selection rules, as specified in the Method section.

According to Eq. (2), the photon transition matrix element presents a dominant term independent of **K** when the transition is allowed. On the contrary, the contribution proportional to **K** must be considered only for a forbidden transition. However, the latter is not the main contribution to the transition rate because the constant term for allowed transitions is by far larger than the terms in **K**, at least in the region of the **K-**space near the critical point that contributes to the integral in Eq. (8). Consequently, by considering 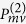, 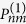, and 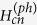 as independent of **K**, the integral on **K** and **k** subject to the restriction satisfying the delta function gives Eq. (9):


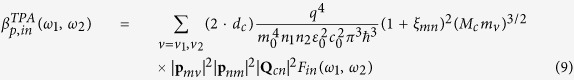


In particular, the term *F*_*in*_(*ω*_1_, *ω*_2_)is calculated as in Eq. (10).


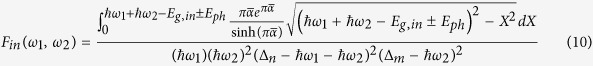


with 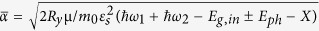, and the subscript (*in*) states for the indirect transitions.

In Eq. (10), the term 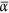 is related to the continuum exciton effect. Moreover, it is worth outlining that the previous equations have been derived under the assumption of parabolic bands. However, our theoretical investigations indicate that the TPA coefficient with non-parabolic bands, 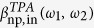, can be well approximated by using Eq. (11).


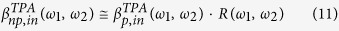


The correction function *R*(*ω*_1_, *ω*_2_) defined as in Eqs (12, 13, 14).


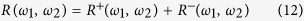



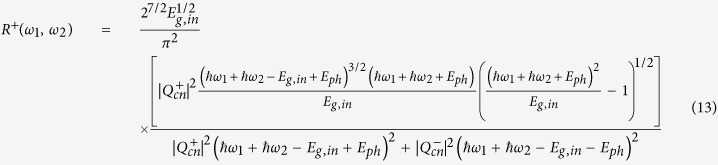



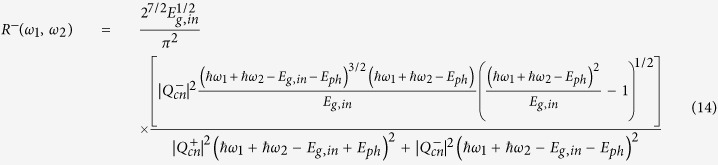


Thus, it is worth noting that Eq. (11) is rigorous in the absence of any continuum exciton influence.

Subsequently, we apply the Kramers-Kronig relationship in order to derive the Kerr nonlinear coefficient (*n*_2_) as in Eq. (15), and using the functional expression of *β*^*TPA*^(*ω*_1_, *ω*_2_)^22^.


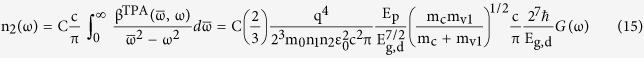


where *G*(*ω*) is the spectral function defined as in Eq. (16), and *C* is a correction coefficient.


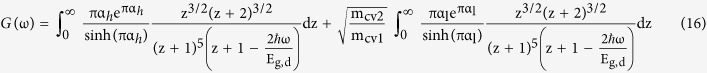


In particular, 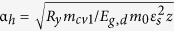, 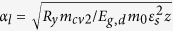, and *z* is the normalized changed variable.

## Results

### Validation

The goal of this section is to validate the theoretical assumptions outlined previously. A combination of simplified two band theory and experimental results available in the literature have been used^17^ in order to compile and plot the key third-order nonlinear optical coefficients of bulk crystalline Si and Ge as a function of the wavelength (1.5–6.7 μm for Si and 2–14.7 μm for Ge). Furthermore, the curve-fitting of the experimental data has been performed to give theoretical predictions in a spectral range where experimental data do not exist. Also, the same procedure has been adopted to predict the third-order nonlinear optical coefficients in the Si_1−x_Ge_x_ alloy. In this context, Ge would provide an interesting case study since direct and indirect two-photon absorption can occur in different spectral regions. Indeed, as evidenced in ref. 24, the Ge degenerate *β*^*TPA*^ is dominated by the direct transitions up to 3.17 μm. On the contrary, experimental observations indicate that the optical intensity attenuation is attributed exclusively to the TPA process involving the indirect band gap in the wavelength range of 3.2–3.6 μm. Thus, the Ge bulk can be considered as test-bench or reference to validate the physical assumptions adopted in the modelling in the cases of direct and indirect transitions.

The Ge physical parameters used in our simulation are detailed in the Method section. Furthermore, Sellmeier’s equations for Ge bulk layer^25^ have been used to take into account the chromatic dispersion of the material in simulations.

**Figure 2:**
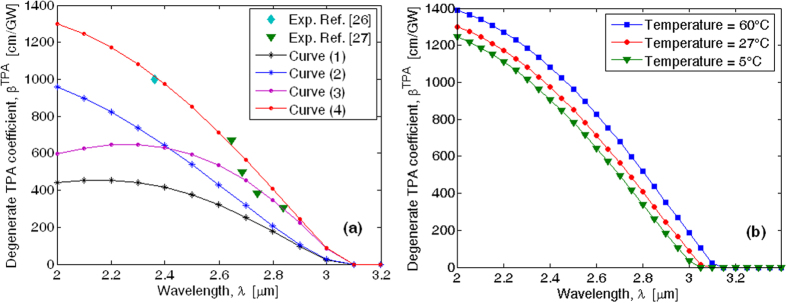
(**a**) Spectra of the degenerate two-photon absorption of Germanium induced by direct transitions. Curve (1), Parabolic bands without continuum exciton influence. Curve (2), Nonparabolic bands without continuum exciton influence. Curve (3), Parabolic bands with continuum exciton influence. Curve (4), Nonparabolic bands with continuum exciton influence; (**b**) Spectra of the degenerate two-photon absorption for different temperature values.

Figure 2(a) shows the degenerate TPA coefficient spectra induced by the direct transitions. In particular, a number of curves have been plotted according to different theoretical assumptions, i.e., Curve (1) parabolic bands and no exciton influence, Curve (2) nonparabolic bands and no exciton influence, Curve (3) parabolic bands with continuum exciton influence, Curve (4) nonparabolic bands with continuum exciton influence. For comparison, the experimental measurements are also included into the plot.

The plot also evidences that the simplified model (i.e., Fig. 2(a), Curve (1)) strongly underestimates the TPA coefficient especially when shorter wavelengths are explored. Thus, in the framework of parabolic bands, also the inclusion of the light hole contribution is not enough to estimate correctly the TPA spectrum. In addition, the nonparabolicity and continuum exciton effects induce an increasing of the TPA coefficient up to 2.25 and 2.5 times, respectively, with respect to the simplified model, also depending on the operating wavelength. In particular, when *λ* > 2.6 μm the continuum exciton influence dominates on the nonparabolicity effect. The opposite trend can be observed when *λ* < 2.6 μm. Therefore, the simultaneous inclusion of both effects allows a good agreement between the theoretical estimations and experimental results (see Fig. 2(a), Curve (4)). It is worth outlining that fitting parameters have not been used in our simulations. Moreover, differently from the fitting procedure proposed in ref. 17, where the numerical results overestimate the measurements in ref. 26 and underestimate the measurements in ref. 27, our predictions seem to match better the aforementioned experimental data with the advantage of gaining physical information on the influence of different effects involved in the direct TPA process.

The spectrum of the degenerate TPA coefficient in presence of nonparabolic and continuum exciton effects is plotted for different temperature values in Fig. 2(b). The simulations have been performed considering the temperature influence on the refractive index^25^ and on the direct energy gap using the relationship^28^


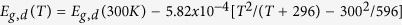


Moreover, considering the identity *E*_*p*_ = *m*_0_*E*_*g*,*d*_/*m*_*c*_^18^, we have also included the temperature effect on the Kane energy and then on the photon element matrix (|***p***_*ab*_|^2^). The plot clearly indicates an increase of both the TPA coefficient and the direct wavelength cut-off as a function of the temperature as mainly induced by the reduction of the direct energy gap.

At this stage, we test the physical model for the estimation of dispersion of phonon- assisted two-photon absorption (see Eqs (9, 10, 11, 12, 13, 14)). The analysis of the dispersion of the two photon absorption and Kerr nonlinearity of indirect semiconductors has been proposed in ref. 22, assuming only the forbidden-forbidden transitions. Recently, an upgraded model has also been proposed in ref. 29, where the authors incorporate the allowed-forbidden and allowed-allowed transitions showing that the latter process dominates the *β*^*TPA*^ coefficient. A similar approach has been followed in refs 17,30, where *β*^*TPA*^ is assumed to have the spectral dependence as in ref. 29, but corrected by a material-dependent fitting coefficient in order to match the experimental data.

**Figure 3:**
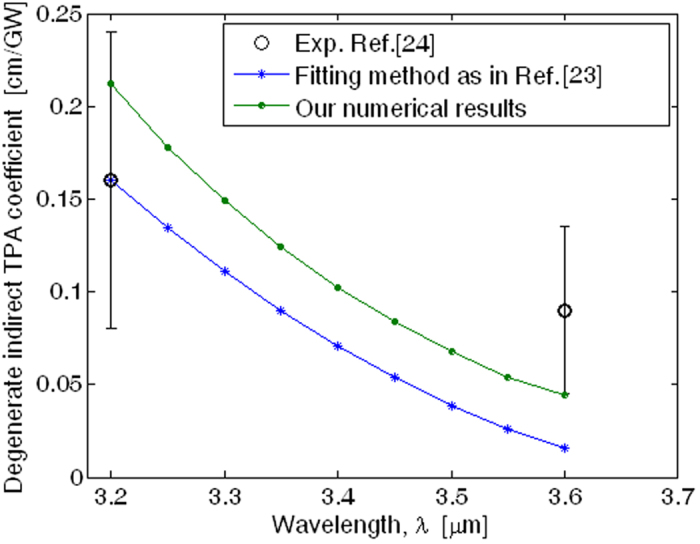
Phonon-assisted degenerate two-photon absorption spectrum of Germanium.

The proposed model with well-defined parameters and physical assumptions, as described in the Method section, and where both nonparabolicity and continuum exciton effects are included, causes the 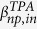 coefficient to have a spectral dispersion as plotted in Fig. 3. Additionally, both the numerical TPA coefficient spectrum obtained with the fitting procedure as in refs 23,30 and the experimental measurements given in ref. 24 have been included in the plot for comparison.

Some comments on the experimental results reported in Fig. 3(a) are worth making. In ref. 24, authors outlined that the obtained indirect TPA coefficient is subject to a few caveats. One is due to the uncertainty in the beam area at the sample. Indeed, a beam diameter variation of 10% can result in a derived TPA coefficient variation of 50%. The second major caveat is due to the uncertainty in the free-carrier absorption cross sections. Authors have reported that a cross section variation of 10% induces a final measurement variation as high as 15%. For these reasons, we have also included the 50% error bar in Fig. 3.

The plot reveals that our predictions are in agreement with the experimental data. Moreover, by comparing our calculations and the fitting approach (see the blue curve in Fig. 3), it is possible to observe an improvement of the TPA spectrum estimation around the wavelength of 3.6 μm. This aspect can be justified as a result of the continuum exciton influence and the nonparabolicity effect induced by the *R* function (see Eq. (11)). Consequently, our modelling overestimates the 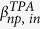 coefficient with respect to the fitting approach, causing the theoretical prediction at a wavelength of 3.6 μm to be barely inside the error bar of 50%, where the fitting approach induces a prediction out from the error bar. As a result, our simulations are only slightly better than the fitting model. However, since the aim of this section is to validate our assumptions and considering the fitting procedure as an accuracy reference, we can conclude that our physical model is consistent and suitable to be applied to the ternary alloy. Indeed at this step, some additional comments are noteworthy on the motivations for the validation of the proposed physical model used to estimate the direct and indirect TPA coefficients. Indeed, the need to ensure a physical interpretation of the nonlinear absorption processes becomes very crucial in the absence of any measurement references. On one hand, the using of a fitting procedure as reported in refs 17,22,23,29,30 represents an efficient and quick tool to estimate the nonlinear coefficients in wavelength regions where experimental data exist only in limited wavelength segments. On the other hand, this approach is unusable in the case of new technology platforms (i.e., SiGeSn alloy, core of this work) for which experimental measurements have not been carried out in any wavelength range. In this sense, the previous analysis represents a fundamental step in order to evaluate if the physical effects considered in our model together with the selection rules and the material parameters (i.e band energy, refractive index, phonon energy, matrix element, etc.) are sufficient to realize TPA predictions with a good accuracy (at least equal to the fitting procedure).

**Figure 4:**
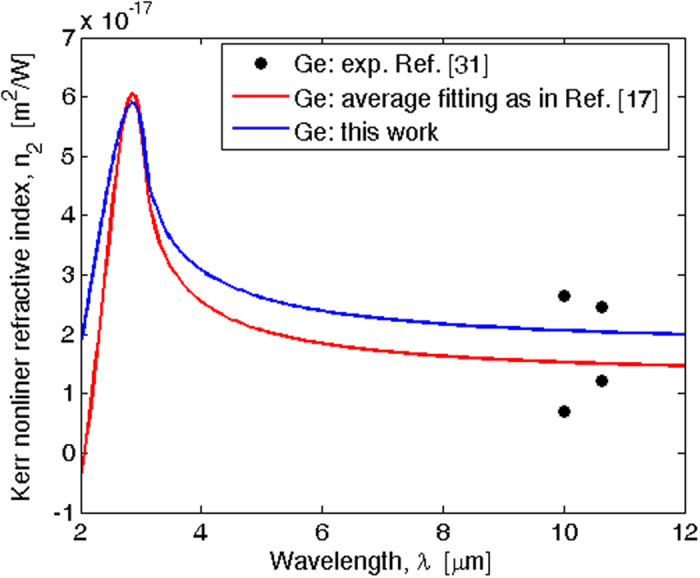
Kerr nonlinear refractive index spectra for Ge material.

Finally, in Fig. 4 we show the Kerr refractive index (*n*_2_) as a function of the wavelength for the Ge material, as obtained by means of Eqs (15 and 16) and assuming the constant *C* = 0.025. The experimental data of^31^, and the average fitting proposed in ref. 17, are also included in the plot for comparison reasons.

In conclusion, the previous analysis leads us to conclude that the physical model adopted is very useful to predict the wavelength dispersion of the direct and indirect TPA processes as well as the wavelength dependence of the Kerr refractive index.

### Third order nonlinearities in Ge_1−x−y_Si_x_Sn_y_ alloy

In this section, we use the physical model to predict, initially, the direct and phonon-assisted TPA coefficients of Ge_1−x−y_Si_x_Sn_y_ ternary alloy. It is worth outlining that in the presence of a few experimental data for Ge, any simplification for the adopted band structure could be compensated by introducing an opportune fitting coefficient. On the contrary, the lack of experimental data, as for the Ge_1−x−y_Si_x_Sn_y_ alloy, must require a more precise band structure information. Thus, we could perform our calculations without any fitting coefficient applying rigorously the selection rules as induced by semiconductor symmetry and evaluating the alloy parameters (i.e band energy levels, matrix element, phonon energy, etc.) as obtained from the values of each semiconductor constituting the alloy, and derived from the realistic band structures (see Method section for details).

However, as outlined in refs 1,12 a better estimation for the energy-band gaps at the conduction *Γ*, *L*, and *X* points of the unstrained Ge_1−x−y_Si_x_Sn_y_ ternary alloy can be achieved as in Eq. (17) by using the quadratic polynomials including the significant bowing effects (*b*^(n)^).


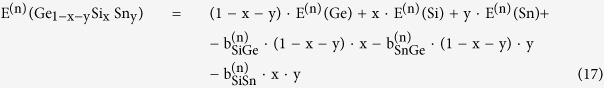


In Eq. (22), *n* = *Γ*, *L*, *X* and the bowing parameter values are given in ref. 1.

Although Eq. (17) is valid in the complete ranges of *x* and *y*, high values of Si and Sn fractions represent a problem for actual epitaxy processes. In this sense, we limit our analysis to the cases of *x* and *y* values within 15% and 10%, respectively.

**Figure 5:**
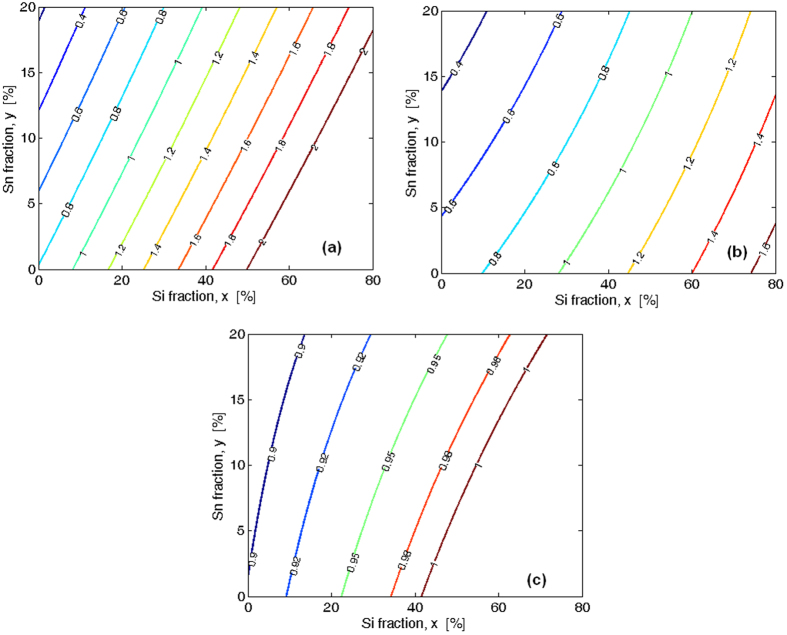
Ge_1−x−y_Si_x_Sn_y_ conduction-band energies of unstrained alloy in eV as a function of the Si and Sn fractions; (**a**) *Γ* point. (**b**) *L* point. (**c**) *X* point.

In Fig. 5(a–c) we show the level curves for the band conduction energies as a function of the Si and Sn fractions at the *Γ*, *L*, and *X* valleys, respectively.

The plot indicates that the energy of the *L* band is always smaller than that of *X* valley for all the *x* and *y* values considered. Moreover, the unstrained Ge_1−x−y_Si_x_Sn_y_ ternary alloy works as an indirect bandgap semiconductor for all the *x* values considered if *y* = 5%, or for *x* > 1% in the case *y* = 10%. Conversely, for *x* < 1% and *y* = 10% the unstrained Ge_1−x−y_Si_x_Sn_y_ ternary alloy works as a direct bandgap semiconductor.

**Figure 6:**
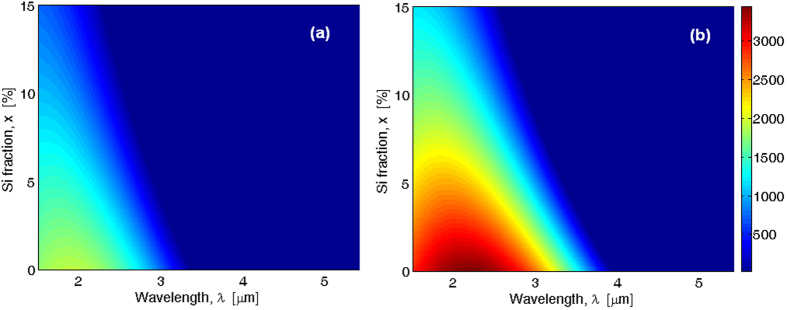
Direct degenerate two-photon absorption coefficient of GeSiSn versus the wavelength and Si fraction. (**a**) *y* = 2% (**b**) 5%.

The plots in Fig. 6(a,b) show the direct TPA coefficient (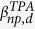) versus wavelength and Si-fraction in the cases of two values of Sn-fraction, i.e., *y* = 2% (Fig. 6(a)), and *y* = 5% (Fig. 6(b)).

**Figure 7:**
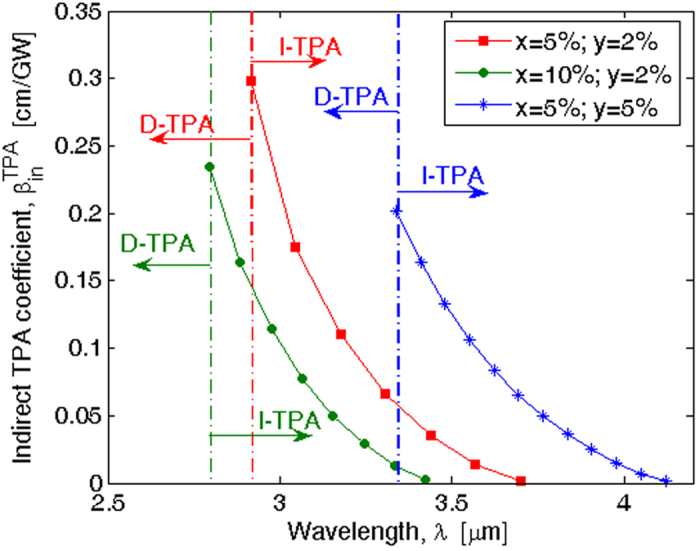
Phonon-assisted degenerate two-photon absorption coefficient spectra of GeSiSn for different values of the Si and Sn fractions.

As expected, because the TPA process is stronger in Ge than in Si, the Ge_1−x−y_Si_x_Sn_y_ response presents 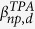 values that are significantly higher than in Si. This will inhibit the behavior of possible photonic devices based on the ternary alloy in certain wavelength regions, generally a near-infrared region. In particular, if *x* = 5%, the 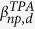 coefficient assumes a maximum value of about 1432 cm/GW (at *λ* = *λ*_*peak*_ = 1.672 μm) and 2422 cm/GW (at *λ* = 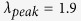
μm) for *y* = 2% and 5%, respectively. Moreover, *λ*_*peak*_ shifts towards shorter values by increasing the Si-fraction up to 15%. In addition, Fig. 6(a,b) indicate that both 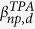 and cut-off wavelength (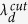) decreases occur as a function of both increasing Si and decreasing Sn fractions. For instance, we estimate that the 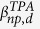
coefficient drops from 2422 cm/GW for *x* = 5%, and *y* = 5% to 484.8 cm/GW in case of *x* = 15%, and *y* = 2% at 1.9 μm. For the same *x*-*y* combinations, the cut-off wavelength moves from 3.34 to 2.29 μm. Consequently, by staying inside of the *x*-*y* epitaxy constraints, it is possible to choose appropriately the Si and Sn fractions in order to select operative wavelength windows where the photonic devices based on the Ge platform cannot operate due to the very high values of TPA (see Fig. 2). Indeed, the TPA is generally strong in the near infrared but drops significantly with increasing the wavelength and vanishes at 2.2 μm in Si, at 3.2 μm in Ge (see Fig. 2), and vanishes in the range 2.29–2.56 μm in Ge_1−x−y_Si_x_Sn_y_ in the case of the direct bandgap TPA process. However, an evaluation of the phonon-assisted two photon absorption process is needed to estimate the effective value of the TPA cut-off. Thus, assuming *y* = 2% and 5% and *x* changing up to 10%, the indirect bandgap of the Ge_1−x−y_Si_x_Sn_y_ alloy is induced by the *L* valley (see Fig. 5). In this context, we can guess that in the ternary alloy only LO phonons are involved in the indirect TPA process (see Method section for details). According to the theory described, Fig. 7 shows the degenerate indirect TPA coefficient, 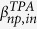, as a function of the wavelength for different values of Si and Sn fractions.

In Fig. 7, the vertical lines individuate the regions where the direct (D-TPA) and indirect (I-TPA) TPA processes act as a function of each value of *x* and *y*. Additionally, it is evident that the 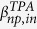 coefficient assumes not negligible values if compared with Si in the near-infrared spectrum. As a result, the effective cut-off wavelength must be evaluated by referring to the curves plotted in Fig. 7, with that wavelength being equal to 3.7, 3.42, and 4.12 μm when (*x* = 5%, *y* = 2%), (*x* = 10%, *y* = 2%) and (*x* = 5%, *y* = 5%), respectively. Similarly, the effective cut-off drops to 3.15 μm assuming (*x* = 15%, *y* = 2%).

**Figure 8:**
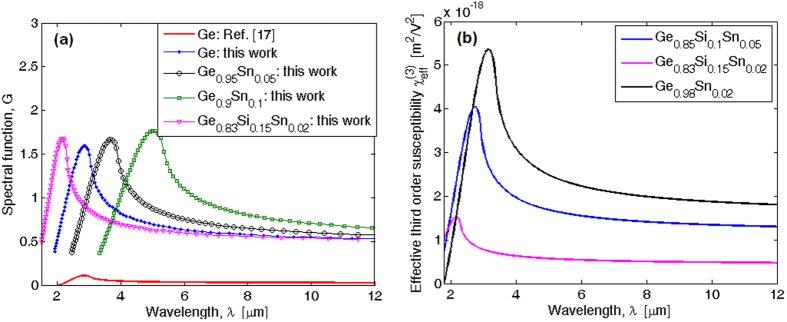
(**a**) Spectral function for different GeSiSn alloy compositions. (**b**) Effective third order susceptibility spectrum for different GeSiSn alloy compositions.

In Fig. 8(a), we show the spectral function (*G*) (see Eq. (16)) as a function of the wavelength for different alloy compositions. The spectral function evaluated as in ref. 17 is also reported for comparison. Several comments are noteworthy. The numerical simulations obtained by Eq. (16) show larger values for both the G function and the peak width than the spectrum proposed in ref. 17, as induced by the continuum exciton and non-parabolicity effects. Moreover, the spectral peak for the Ge_1−y_Sn_y_ alloy shifts towards larger wavelength as the Sn content *y* increases. Conversely, the opposite trend in peak location is obtained for Ge_1−x−y_Si_x_Sn_y_ ternary alloy if the Si and Sn fractions are increased and decreased, respectively. Finally, Fig. 8(b) shows the effective third order susceptibility, defined as 
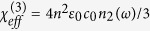
, versus the wavelength for different alloy compositions. Moreover, in the simulations we have assumed than the constant *C*, adopted for the Ge bulk, holds the same order of magnitude for Ge_1−x−y_Si_x_Sn_y_ alloy having relatively low values of *x*, and *y*. The plot indicates that the 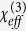 coefficient can be estimated at 10.6 μm as 1.33 × 10^−18^, 4.81 × 10^−19^, and 1.84 × 10^−18^ m^2^/V^2^ for Ge_0.85_Si_0.1_Sn_0.05_, Ge_0.83_Si_0.15_Sn_0.02_, and Ge_0.98_Sn_0.02_ respectively, with the peak value (at shorter wavelength) being ~2.5 *x* higher.

The present analysis indicates that the Ge_1−x−y_Si_x_Sn_y_ is a good candidate for nonlinear optical applications, such as FWM, continuum generation, and wavelength conversion which can be integrated together with passive and active functionalities in order to realise complex optoelectronic chips. For the various *χ*^(3)^ and *n*_2_ four-wave applications just cited, the wavelength of the pump laser would be chosen to be at a wavelength where nonlinear loss is very low. That wavelength selection can be accomplished by examining both the *χ*^(3)^ spectral response in Fig. 8 and the nonlinear absorption spectrum in Fig. 7 (or equivalent).

Finally, we believe that the physical model proposed gives realistic theoretical predictions and a comprehensive physical overview of the Ge_1−x−y_Si_x_Sn_y_ ternary nonlinear nonresonant properties over a wide wavelength range, from near-IR to mid-IR. Additionally, although fitting parameters have not been used in the simulations of the TPA processes, the model predictions could be further improved by fitting the experiments on the third order nonlinearity. Indeed, experimental measurements could be used to better set the values of physical parameters such as |**p**_*mv*_|^2^, |**p**_*nm*_|^2^, |**Q**_*cn*_|^2^, which have been numerically estimated in this work by means of the linear interpolation formula (see Eq. (21) in “Method” Section). We believe that the first-principles theory presented here in above sections has generality in the sense that it can be applied readily to group IV alloys containing carbon, and to semiconductors in groups III-V and II-VI.

## Conclusions

In this paper, a mathematical modeling based on a physical approach has been implemented for investigating the spectrum of the two photon absorption induced by direct and indirect transitions in the unstrained Ge_1−x−y_Si_x_Sn_y_ alloy. The proposed model has been validated by comparing our predictions with the experimental measurements listed in the literature for the Ge material.

As a result, a good agreement has been achieved, also demonstrating that the correct estimation of the TPA coefficient should include the effects of nonparabolicity and the influence of the continuum exciton in both cases of direct and indirect transitions. Therefore, the Ge testing analysis has been used as a starting point for the estimation of the TPA processes in the Ge_1−x−y_Si_x_Sn_y_ ternary alloy. Direct TPA coefficient has been evaluated as a function of the wavelength for several choices of Si, and Sn concentrations in order to figure out the wavelength ranges where the photonic device behavior could be inhibited by the strong TPA effect. Moreover, the effective TPA cut-off wavelength (where TPA vanishes) has been determined as a function of different alloy compositions by means of numerical simulations of the phonon-assisted two photon absorption process. The modelling results have evidenced that the longitudinal optical or transverse acoustic phonons are involved into the process for the ternary alloy in which the indirect bandgap is induced by the *L* or *X* CB valleys, respectively. Finally, the intensity-dependent Kerr refractive index has been calculated as a function of the wavelength and of different Si and Sn concentrations. The numerical predictions have shown that the unstrained Ge_1−x−y_Si_x_Sn_y_ ternary alloy can be considered as a very good candidate for nonlinear optical (four-wave mixing) applications since it can guarantee a Kerr effect much larger than that Si in the mid-IR region, for example, at pump wavelengths longer than 3.15 μm.

## Methods

The proposed physical model has been previously validated by using experimental data of Ge indirect TPA coefficient, 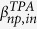
24, Ge degenerate TPA coefficient, *β*^*TPA*^ 26,27, and Ge Kerr nonlinear refractive index, *n*_2_^31^.

As it is evident from the equation set proposed in the Background Section, the direct and indirect TPA coefficients can be uniquely evaluated if the material parameters such as direct and indirect bandgap energy, intermediate state energy and electron momentum matrix element are known. In this context, the **k·p** method has been extremely successful in establishing relationships between various band parameters of semiconductors. In particular, as demonstrated in ref. 32, very good results about the energy bands of germanium and silicon, throughout the entire Brillouin zone, have been obtained by diagonalizing a **k·p** Hamiltonian referred to 15 basis states at **K** = 0. As outlined by Cardona *et al*.^32^, numerical calculations in terms of energy levels and matrix elements have shown a very good agreement with the experimental data from the cyclotron resonance. In this sense, the parameters evaluated in ref. 32 are used in this work for characterizing the germanium and silicon bulk materials. It is worth outlining that our investigations indicate that weak changes in the values proposed in^32^ do not affect the results and physical considerations described in the previous sections. In Table 1, we summarize the fundamental coefficients for Ge semiconductor evaluated at room temperature and used in our simulations.

Moreover according to^32^, the Ge electron momentum matrix element,|**p**_*cv*_|^2^, has been assumed equal to 1.8658 × 10^−48^ Kg·J. Furthermore, the value of |**p**_*cv*_|^2^ induces a Kane energy *E*_*p*_ = 25.56 eV in fairly good agreement with the values listed in other different works^11^,^33^.

More sophisticated is the case of the indirect TPA process. Indeed not only the knowledge of the values of 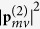, 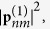
and |**Q**_*cn*_|^2^ (see Eq. (9)) is fundamental in order to calculate the TPA coefficient, but it is crucial to have physical indications about the intermediate states *n* and *m* involved in the phonon-assisted nonlinear absorption. In this sense, some comments on the selection rules are worth specifying.

Preliminary qualitative results can be obtained as a function of the semiconductor symmetry without any explicit calculation of the semiconductor wave functions. Indeed, it is possible to estimate if the matrix elements are zero or are different from zero and then to find selection rules which exclude some of the intermediate states and some of possible phonons taking part in the indirect TPA processes described by Eqs (8. For instance, Eq. (9) states that the *β*^*TPA*^(*ω*_1_, *ω*_2_) coefficient is directly proportional to the product 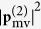 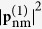, as a result the semiconductor symmetry could induce the two matrix elements to be zero if an exchange among the two intermediate states is applied. In this context, it is convenient to express the selection rules using the general method of group theory in order to take into account the crystal symmetry^34^. In the case of Ge and considering the schematic diagram in Fig. 1, the top of the valence state, the lowest conduction state, and the nearest intermediate states, are denoted (BSW notation^34^) by Γ′_25_, *L*_1_ (indirect bandgap), Γ′_2_ (direct bandgap), Γ_15_, and Γ_1_, respectively. Also, taking into account the symmetry of the photon transition (Γ_15_), and following the group theory, the allowed states from optical transition from the initial state (Γ′_25_) are reported in Eq. (18), where only Γ_15_ gives an allowed transition to Γ_1_^34^.


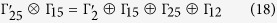


Similarly, the group theory confirms Eq. (19).


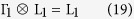


In conclusion, in the case of Ge the most favorable two-photon indirect transitions involve only the following steps: 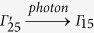, 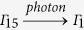 (see Eq. (15)), and 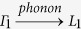, in which only the longitudinal optical (LO) phonons are allowed (see Eq. (19)). Thus, by referring to Eq. (18), the exchange among 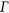
_15_, and 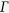
_1_ is forbidden, resulting in *ξ*_*mn*_ = 0 (see Eq. (9)). Thus in the plot of Fig. 3, we have assumed |**p**_*mv*_|^2^ = 1.5 × 10^−48^ Kg·J and |**p**_*nm*_|^2^ = 1.16 × 10^−48^ Kg·J, according to **k·p** calculations^32^.

Moreover, according to^23^, the |**Q**_*cn*_|^2^ parameter for the electron-LO phonon scattering is given by Eq. (20).


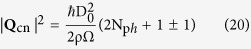


In Eq. (20), N_p*h*_ is the photon occupation number given by Eq. (21).


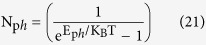


In Eq. (20), *D*_0_ and *ρ* represent the optical deformation potential and the semiconductor density, respectively, as listed in Table 1 in the case of Ge. The term *Ω* indicates the phonon frequency, and the ± signs correspond to phonon absorption and emission, respectively.

The considerations before mentioned for the Ge also holds for the Ge_1−x−y_Si_x_Sn_y_ ternary alloy, if the energy parameters are opportunely evaluated.

In order to obtain most of the parameters for the Ge_1−x−y_Si_x_Sn_y_ material system, linear interpolations among Si, Ge, and α-Sn parameters are used. The linear interpolation formula for the generic physical parameters, A(Ge_1−x−y_Si_x_Sn_y_), with the exception of the bandgaps, is given by Eq. (22)^11^.


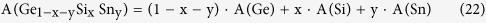


The physical parameters used for our investigations at room temperature are listed in Table 2.

Thus using the matrix element values for each bulk material (Ge, Si, and Sn) and applying Eq. (21), we estimate that if the Si concentration, *x*, ranges from 5% to 15%, the photon matrix element alloy |**p**_cv_|^2^ for the direct transitions changes ranging from 1.8485 × 10^−48^ to 1.8196 × 10^−48^ Kg·J, and from 1.845 × 10^−48^ to 1.8162 × 10^−48^ Kg·J for *y* = 2% and 5%, respectively. These values have been used in order to obtain Fig. (6).

Moreover for the *x* and *y* values for which the *L* band is always smaller than that of *X* valley, we can consider as valid Eqs (18, 19, 20) and then we can guess that in the ternary alloy presents the same physical behavior of the Ge bulk. Therefore, the most favorable two-photon indirect transitions involve only the following steps: 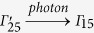, 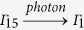, and 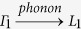, where only LO phonons are allowed. Under this physical assumption, the results of Fig. 7 have been performed by estimating, through Eq. (22)**p**_*mv*_|^2^ = 1.484 × 10^−48^, 1.468 × 10^−48^, 1.485 × 10^−48^ Kg·J and |**p**_nm_|^2^ = 1.158 × 10^−48^, 1.156 × 10^−48^, 1.159 × 10^−48^, Kg·J for the (*x, y*) combinations equal to (*x* = 5%, *y* = 2%), (*x* = 10%, *y* = 2%) and (*x* = 5%, *y* = 5%), respectively. In addition with reference to the band diagram of the Ge_1−x−y_Si_x_Sn_y_ ternary alloy, we can reasonably assume that the degeneracy factor d_c_ in Eq. (9) is the same as in the Ge material (*d*_*c*_ = 4).

Conversely, if the indirect bandgap of the Ge_1−x−y_Si_x_Sn_y_ ternary alloy is mainly influenced by the *X* conduction valley (i.e., *y* = 5%, and *x* > 37% or *y* = 10%, *x* > 48%), we believe that the physical model must be modified to take into account the different phonon nature involved into that process. According to the group theory, the 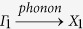 transition is allowed for phonons of symmetry so that the relationship Γ_1_⊗*X*_1_ = *X*_1_ is satisfied. As a result, only the transverse acoustic (TA) phonons can be involved in the indirect TPA process.

In this context, the |*Q*_*cn*_|^2^ parameter in Eq. (9) for the electron-TA phonon scattering is given by Eq. (23)^23^.


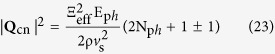


In Eq. (23), where the terms *v*_*s*_ and *Ξ*_*eff*_ represent the acoustic velocity and effective deformation potential.

## Additional Information

**How to cite this article**: De Leonardis, F. *et al*. Dispersion of nonresonant third-order nonlinearities in GeSiSn ternary alloys. *Sci. Rep.* **6**, 32622; doi: 10.1038/srep32622 (2016).

## Figures and Tables

**Table 1:** Germanium room temperature physical parameters used in simulations.

Parameters	Values
*E*_*g*,*d*_ [eV]	0.7985
*E*_*g*,*in*_ [eV]	0.664
Δ_*m*_ [eV]	3.15
Δ_*n*_ [eV]	7.768
*E*_*ph*_ [eV]	0.037
*m*_*v*1_/*m*_0_	0.28
*m*_*v*2_/*m*_0_	0.044
*m*_*c*_/*m*_0_ (Γ valley)	0.038
*m*_*t*_/*m*_0_ (L valley)	0.0807
*m*_*l*_/*m*_0_ (L valley)	1.57
*d*_*c*_	4
*D*_0_ [eV m^−1^]	7.8 × 10^10^
*ρ* [Kg/m^3^]	5.3267 × 10^3^

**Table 2:** Parameters at room temperature of Si, Ge and α-Sn.

Parameters	Si	Ge	α-Sn
*m*_*c*_/*m*_0_ (*Γ* valley)	0.528	0.038	0.058
*m*_*t*_/*m*_0_ (*L* valley)	0.133	0.0807	0.075
*m*_*l*_/*m*_0_ (*L* valley)	1.659	1.57	1.478
*m*_*v*1_/*m*_0_	0.49	0.28	0.3
*m*_*v*2_/*m*_0_	0.16	0.044	0.038
*E*(Γ) [eV]	3.302	0.8113	−0.4102
*E*(*L*) [eV]	2	0.7013	0.14
*E*(*X*) [eV]	1.2021	0.9013	0.9102
*E*_*p*_ [eV]	21.6	25.26	24.0
Δ_*m*_ [eV]	3.4286	3.15	~3
Δ_*n*_ [eV]	7.07	7.768	~8
density [gr/cm^3^]	2.328	5.323	7.287
