# Corrigendum: Dispersion of nonresonant third-order nonlinearities in GeSiSn ternary alloys

**DOI:** 10.1038/srep34968

**Published:** 2016-10-10

**Authors:** Francesco De Leonardis, Benedetto Troia, Richard A. Soref, Vittorio M. N. Passaro

Scientific Reports 6: Article number: 32622; 10.1038/srep32622 published online: 10 10 2016; updated: 10 10 2016.

This Article contains errors. In Equation 13,
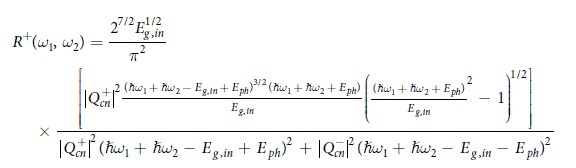


should read:
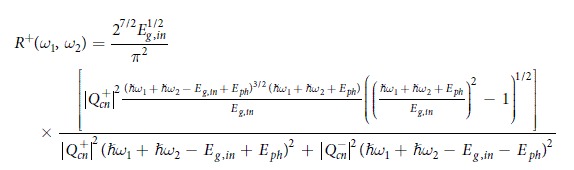


In Equation 14,
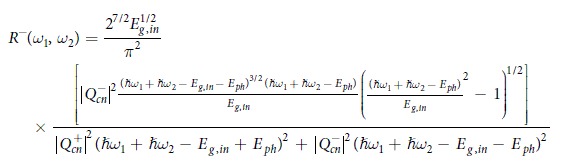


should read:
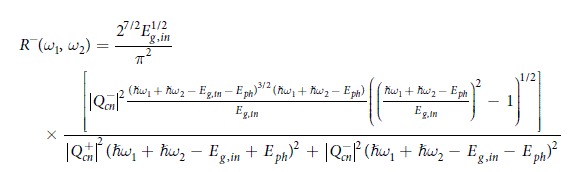


In the Methods section,

“As a result, only the transverse acoustic (TA) phonons can be involved in the indirect TPA process.

In this context, the |*Q*_*cn*_|^2^ parameter in Eq. (9) for the electron-TA phonon scattering is given by Eq. (23)^23^”.

should read

“As a result, only the longitudinal acoustic (LA) phonons can be involved in the indirect TPA process.

In this context, the |*Q*_*cn*_|^2^ parameter in Eq. (9) for the electron-LA phonon scattering is given by Eq. (23)^23^”.

